# Molecular changes in bone marrow, tumor and serum after conductive ablation of murine 4T1 breast carcinoma

**DOI:** 10.3892/ijo.2013.2185

**Published:** 2013-11-21

**Authors:** BEATA D. PRZYBYLA, GAL SHAFIRSTEIN, SAGAR J. VISHAL, RICHARD A. DENNIS, ROBERT J. GRIFFIN

**Affiliations:** 1 Department of Radiation Oncology, University of Arkansas for Medical Science, Little Rock, AR;; 2 College of Medicine, University of Arkansas for Medical Science, Little Rock, AR;; 3 Department of Cell Stress Biology and Otolaryngology, Roswell Park Cancer Institute, Buffalo, NY;; 4 Geriatric Research Education and Clinical Center, Central Arkansas Veteran Healthcare System, Little Rock, AR, USA

**Keywords:** thermal ablation, 4T1 tumors, bone marrow, gene expression, SDF1, HSP27, HSP70, *Esele*, *Cxcl12*, *Fgf2*, *Lifr*, cytokines

## Abstract

Thermal ablation of solid tumors using conductive interstitial thermal therapy (CITT) produces coagulative necrosis in the center of ablation. Local changes in homeostasis for surviving tumor and systemic changes in circulation and distant organs must be understood and monitored in order to prevent tumor re-growth and metastasis. The purpose of this study was to use a mouse carcinoma model to evaluate molecular changes in the bone marrow and surviving tumor after CITT treatment by quantification of transcripts associated with cancer progression and hyperthermia, serum cytokines, stress proteins and the marrow/tumor cross-talk regulator stromal-derived factor 1. Analysis of 27 genes and 22 proteins with quantitative PCR, ELISA, immunoblotting and multiplex antibody assays revealed that the gene and protein expression in tissue and serum was significantly different between ablated and control mice. The transcripts of four genes *(Cxcl12, Sele, Fgf2, Lifr)* were significantly higher in the bone marrow of treated mice. Tumors surviving ablation showed significantly lower levels of the *Lifr* and *Sele* transcripts. Similarly, the majority of transcripts measured in tumors decreased with treatment. Surviving tumors also contained lower levels of SDF-1α and HIF-1α proteins whereas HSP27 and HSP70 were higher. Of 16 serum chemokines, IFNγ and GM-CSF levels were lower with treatment. These results indicate that CITT ablation causes molecular changes which may slow cancer cell proliferation. However, inhibition of HSP27 may be necessary to control aggressiveness of surviving cancer stem cells. The changes in bone marrow are suggestive of possible increased recruitment of circulatory cancer cells. Therefore, the possibility of heightened bone metastasis after thermal ablation needs to be further investigated and inhibition strategies developed, if warranted.

## Introduction

Thermal ablation therapy destroys cancer cells by delivering electromagnetic or acoustic energy [e.g. radiofrequency (1), laser (2), or focused ultrasound (3)] that is converted to heat in the target tissue. The therapy raises the temperature of the ablation site to between 56 and 100°C which results in irreversible damage by coagulation necrosis. Preclinical study with conductive interstitial thermal therapy (CITT) (4), (5) has demonstrated decreased metastasis in rabbits with VX2 tumors (6) and the reduction of hypoxia in tumor tissue surviving partial CITT ablation of murine breast carcinoma (7). Investigating the molecular changes associated with CITT and with other thermal ablation therapies may identify biomarkers that facilitate development of methods for monitoring and predicting the potential for therapy-associated enhancement of tumor re-growth, and metastasis.

Histological analysis of thermal ablation lesions in animal models has revealed three regions. The central region is adjacent to the ablation (i.e. area of probe insertion, or focus of ultrasound) and contains necrotic tumor tissue. Massive tumor debris appears to result in release of immunogenic factors (8). Adjacent to necrotic area is a coagulation margin that defines a sharp transition zone between necrotic and viable tissue. This highly hypoxic region was shown to be a site of accelerated tumor re-growth after radiofrequency ablation (9). A third region is more distant from the center of heat delivery and contains viable tumor tissue. In this region, we observed decreased hypoxia 72 h after CITT ablation (7). Thus, lesions of ablated tumor appear to possess areas with different properties that may have the potential to initiate induction of tumor re-growth (undesirable effect), stimulate antitumor immunity and decrease hypoxia (desirable effects).

Our research is interested in the mechanisms by which thermal tumor ablation affects communication between tumor tissue, bone marrow and sites of metastasis. Recent progress in understanding tumor biology has revealed crucial roles for the bone marrow in carcinogenesis, tumor growth and metastasis. Cytokines and growth factors secreted by tumor into circulation stimulate release of a variety of cells from bone marrow. These molecules contribute to tumor growth, vascularization, and regulation of immune cells in the tumor microenvironment (10,11). However, little is known about the effects of thermal ablation upon the interactions between tumors, bone marrow, and site of metastasis. Thus, characterization of the molecules that potentially mediate these communications in the context of tumor growth and treatment would be an important step towards understanding the mechanisms underlying different outcomes of thermal tumor ablation.

The study presented here reports the preliminary results of quantitative analysis of gene expression and protein levels for serum, marrow and surviving tumor collected 72 h after partial ablation of 4T1 carcinoma in BALB/c mice. The CITT treatment protocol was designed to result in only partial tumor ablation to enable characterization of the remaining surviving tumor tissue. The study hypothesized that tumor ablation would broadly affect gene expression (Table I) and protein (Table II) levels for molecules involved in cell signaling, proliferation, adhesion, angiogenesis and stress response. Candidate molecules were chosen due to evidence in the literature for their role in breast tumor development and spreading, bone marrow homeostasis and tumor responses to thermal stress (12–16).

## Materials and methods

### Growing 4T1 tumors in BALB/c mice and CITT treatment

The 4T1 mammary carcinoma is a syngeneic mouse model of breast cancer. The 4T1 cell line originated from spontaneously grown, metastatic mammary tumor in BALB/c mice. This mammary tumor has many characteristics of human invasive breast cancer (17). The thermal ablation procedures were described in detail in our previous study (7). The use of BALB/c mice (Jackson Laboratories, Bar Harbor, ME, USA) and the experimental protocol was approved by the University of Arkansas for Medical Sciences Institutional Animal Care and Use Committee. The 4T1 cancer cells (2×10^5^) were injected subcutaneously into the right rear leg of each mouse. When tumor reached 10–12 mm in size, the mice were anesthetized with 1–2% isofluorane and a CITT probe, without pin deployment, was inserted in the center of a tumor. Temperatures were monitored with two thermocouples, one next to the tip of the CITT probe and another one, at the periphery of the tumor. Ablation was performed for 10 min with temperature maintained within the range 80–90°C (next to the CITT probe) while the peripheral tumor temperature remained in the low to mid 40°C range. Each mouse had one tumor. Five mice with tumors were ablated and four mice with tumors were used as untreated control.

### Sample preparation

The mice were scarified 72 h after ablation. At this time point necrotic area is well established and the transition zone separating necrotic area from viable perinecrotic zone is clearly visible on tumor sections. The entire tumors were removed immediately after necropsy. One half of the tumor was used to prepare fresh sections in order to record the size of the ablated areas with a viability staining, triphenyltetrazolium chloride (TTC) as previously described (18). The other half of the tumor was used to prepare tissue samples from viable portion of tumors which were cut into pieces ranging from 50–100 mg, frozen in liquid N_2_ and stored at −80°C until use. Femurs and tibiae were carefully cleaned from adherent soft tissue and placed in cold, sterile phosphate buffered saline, followed by transfer to Petri dish filled with Dulbecco’s modified Eagle’s medium (DMEM). The ends of bone were cut off with sterile scissors so that the bone marrow could be flushed with DMEM using a 3cc syringe and 27G needle. The cells suspensions were filtered through 70-mm BD Falcon nylon mesh (Fisher Scientific Inc., Sewanee, GA, USA). Cells were counted (Beckman Coutler Z series System, Hialeah, FL, USA), centrifuged at 1410 rpm for 5 min at 4°C, and re-suspended in serum free DMEM at a concentration 1×10^6^ cells per ml. Aliquots of 1.5 ml of cells were made, centrifuged and the medium was removed so that cell pellets could be frozen in liquid N_2_ and stored at −80°C until use.

### Total RNA isolation and preparation of cDNA

Total RNA was isolated from frozen tumor and bone marrow cell pellets using the RNAqueous-4PCR kit (Ambion/Applied Biosystems, Foster City, CA, USA) according the manufacturer’s instructions. RNA concentration was determined with ND-1000 Spectrophotometer (NanoDrop Technologies, Inc., Wilmington, DE, USA) and RNA integrity was verified with Bioanalyzer (Applied Biosystems). All RNA samples possessed an RNA integrity number (RIN) >7. cDNAs were prepared from 2 *μ*g of RNA using iScript cDNA Synthesis kit (Bio-Rad Laboratories, Inc., Hercules, CA, USA). This kit contains blend of oligo dT and random hexamers primers. The cDNAs were stored at −20°C in 5–10 *μ*l aliquots. A pooled sample of all cDNAs was also prepared to use in primer testing and optimization of standard curves.

### Quantititative RT-PCR

Primers used in the real-time polymerase chain reaction (RT-PCR) were designed using the murine NCBI nucleotides database and Primer Express v3.0 software (Table III). RT-PCR assays were performed according to the manufacturer’s instructions using iTaqSYBR Green Supermix with ROX (Bio-Rad Laboratories Inc.), 384-well plates, and the ABI Prism 7900 Sequence Detection System (Applied Biosystems). For each transcript of interest standard curves were evaluated in order to optimize the primer concentration for maximum reaction efficiency. Standard curves were prepared from five-fold serial dilution of pooled cDNA. Gene expression was normalized to both 18S ribosomal RNA and glyceraldehyde 3-phosphate dehydrogenase (GAPDH). The comparative C_T_ (ΔΔC_T_) method for calculating relative gene expression was used to evaluate differences in levels of transcripts between control group (mice with non-treated tumors) and treated group (mice with ablated tumors) (19). The fold change of transcripts was calculated using DataAssist v3.0 software (Applied Biosystems).

### Quantification of chemokines in serum

Blood was collected by cardiac puncture of anesthetized mice just before euthanasia. Isolated serum was aliquoted for storage in −20°C. Cytokines in serum were quantified with the Cytokine 16-plex panel (Quansys Biosciences, Inc., Logan, UT, USA). This chemiluminescent Quansys Q-plex Array contained IL-1α, IL-1β, IL-2, IL-3, IL-4, IL-5, IL-6, IL-10, IL-12, IL-17, MCP-1, IFNγ, TNFα, MIP-1α, GM-CSF and RANTES antibodies absorbed to each well of a 96-well plate (Table II). The lower level of detection was different for each cytokine and ranged from 0.1 pg/ml (TNFα) to 5.0 pg/ml (MCP-1). Serum samples were tested in triplicate. Data were collected using Q-View Imager and software (Quansys Biosciences Inc.). Results are presented in picograms of cytokine per ml of serum (pg/ml).

### ELISA

Tumor lysates were prepared from frozen tumors by homogenization in diluted Cell Lysis Buffer 10X (Cell Signaling Technology, Inc., Beverly, MA, USA) containing 1 mM PMSF (phenylmethanesulfonyl fluoride). The total protein concentration of lysates was determined with Pierce BCA Protein Assay kit, (Thermo Fisher Scientific Inc., Rockford, IL, USA), a Synergy HT spectrophotometer and the Gene5 software (BioTech Instruments, Winooski, VT, USA). The level of stromal derived factor 1 (SDF-1, also known as chemokine CXCL12) in the lysates was determined using Duo Set ELISA Development kit, according the manu facturer’s instructions (cat # DY460, R&D Systems, Inc., Minneapolis, MN, USA). Samples were assessed in triplicate. The results are presented as picograms of SDF-1 protein per micrograms of total protein content of the tumor lysate.

### Western blotting

Tumor lysates for immunoblotting were prepared as described above for ELISA. Novex Pre-Cast gradient gels (4–20%) and NuPAGE Electrophoresis system (Invitrogen Life Technologies, Inc., Carlsbad, CA, USA) were used to resolve proteins from 8 *μ*g of total protein sample per well. Western transfer onto polyvinylidene difluoride membranes (Amersham, Piscataway, NJ, USA) was performed in an XCell II Blot Module (Invitrogen Life Technologies, Inc.). After protein transfer, membranes were stored at 4°C. For immunoblotting, membranes were incubated in 10% fat-free powdered milk solution for 4 h at room temperature (RT). Primary antibodies were applied for 1 h at RT, the membrane was washed, and the secondary antibody was applied for 1 h at RT. The following primary antibodies were used: monoclonal anti-HIF-1α (cat # NB100-105, Novus Biologicals), polyclonal anti-HSP 27 (cat # sc-1049, Santa Cruz Biotechnology), monoclonal anti-HSP 70 (cat # sc-24, Santa Cruz Biotechnology), monoclonal anti-Ki67 (cat # NBP1-40684, Novus Biologicals) rabbit polyclonal anti-MMP9 (cat # ab38898, Abcam) and anti-actin (1–19) (cat # sc1616, Santa Cruz Biotechnology). The secondary antibodies were: anti-mouse (cat # 170-6516; Bio-Rad Laboratories, Inc.), anti-goat (cat # sc-2020; Santa Cruz Biotechnology) and anti-rabbit (cat # sc-2004, Santa Cruz Biotechnology).

### Statistical analysis

The two-tailed Student’s t-test was used to analyze RT-PCR and ELISA data. Mann-Whitney U test and Fisher’s exact test were used to evaluate cytokines panel results. The software DataAssist v3.0 (Applied Biosystems) and Sigma Plot v11 (Systat Software Inc., San Jose, CA, USA) were applied.

## Results

### Effects on bone marrow gene expression

**Figure 1.**
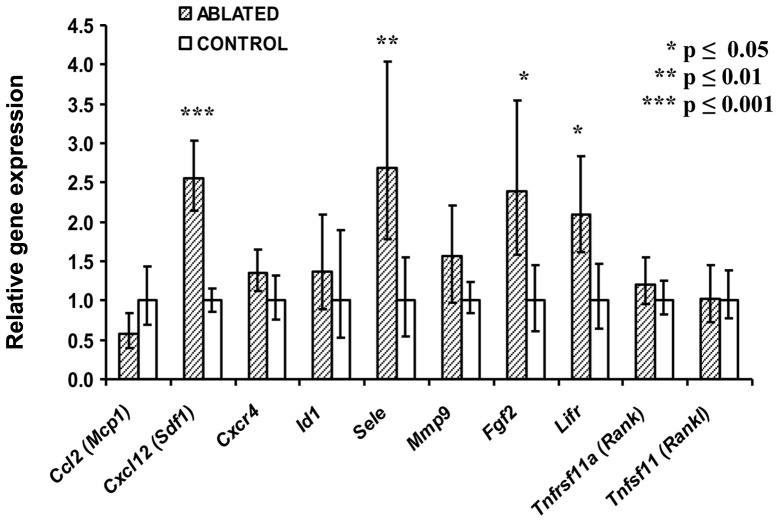
Transcript levels in RNA from bone marrow of BALB/c mice. Quantitative real-time RT-PCR was used to quantify 27 genes of interest in marrow isolated from treated (N=5) and control (N=4) mice 72 h after partial thermal ablation of 4T1 mammary carcinoma, subcutaneously grown in BALB/c mice. Expression data for the genes of interest was normalized to 18S levels. Bars represent the relative expression (RQ) compared between marrows from ablated versus control mice. RQ is calculated as 2^−(ΔΔCT)^ and error bars represent RQ min = 2^−(ΔΔCT+STD)^ and RQ max = 2^−(ΔΔCT-STD)^. Results are shown for 10 of the 27 transcripts quantified by the study.

The effects on the bone marrow of BALB/c mice 72 h after tumor ablation by CITT therapy were measured by quantitative real-time RT-PCR. Marrow RNA was assayed for 27 murine transcripts (Table I). Four transcripts were expressed at significantly higher levels for the mice treated with tumor ablation (N=5) as compared to the control mice (N=4) with untreated tumors (Fig. 1). Transcript levels for genes encoding stromal derived factor 1 *(Cxcl12)*, E-selectin *(Sele)*, leukemia inhibitory factor 1 receptor *(Lifr)* and basic fibroblast growth factor *(Fgf2)* ranged from an average of 2 to 2.5-fold higher (P<0.05) in the treated as compared to the untreated mice. Transcripts of other genes such as chemokine ligand-2 *(Ccl2)* and chemokine receptor-4 *(Cxcr4)* were not affected in bone marrow by tumor ablation (Fig. 1). Four other examples of transcripts unaffected by the treatment are also shown.

### Effects on surviving tumor gene expression

**Figure 2.**
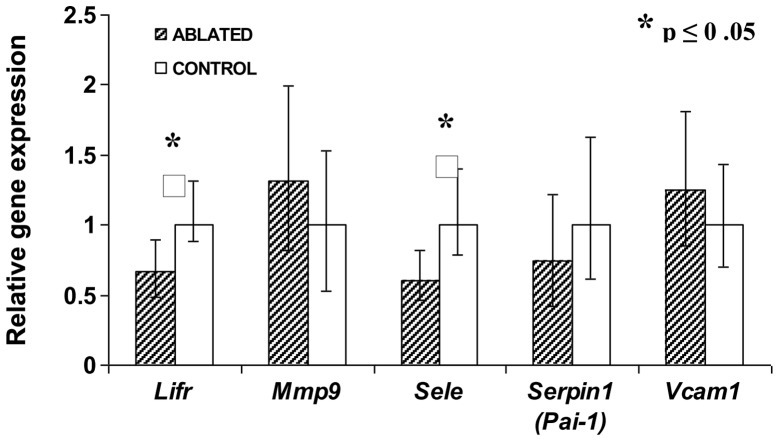
Transcript levels in RNA from viable 4T1 tumors. Quantitative real-time RT-PCR was used to quantify 27 genes of interest in RNA isolated from viable tumor of treated (N=5) and control (N=4) mice 72 h after partial thermal ablation. Expression data for the genes of interest were normalized to 18S levels. Bars represent the relative expression (RQ) compared between marrows from ablated versus control mice. RQ is calculated as 2^−(ΔΔCT)^ and error bars represent RQ min = 2^−(ΔΔCT-STD)^ and RQ max = 2^−(ΔΔCT-STD)^. Results are shown for 5 of the 27 transcripts quantified by the study.

The effects on viable 4T1 tumor surrounding the site of partial ablation were also measured by quantitative real-time RT-PCR. Tumor RNA was assayed for 27 murine transcripts (Table I) for treated (N=5) and control mice (N=4). Two transcripts were expressed at significantly lower levels for the mice treated with tumor ablation (N=5) as compared to the control mice (N=4) with untreated tumors (Fig. 2). Transcript levels of *Lifr* and *Sele* genes for treated tumors was only ∼60% as high as the control tumors (P<0.05). Transcripts of other genes such as matrix metallopeptidase-9, serpin-1 and vascular cell adhesion molecule-1 were not affected in tumor tissue that survived ablation (Fig. 2).

### Effects on surviving tumor protein level

**Figure 3.**
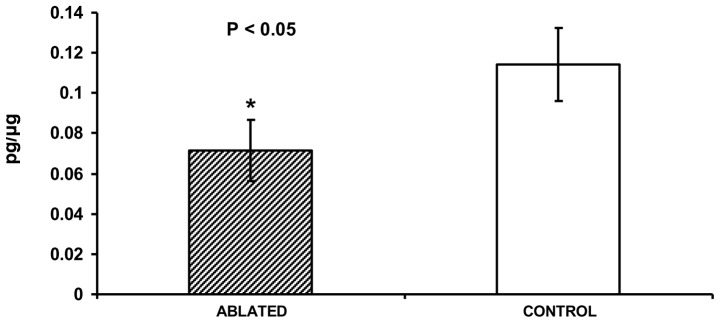
Protein levels for SDF1 in 4T1 tumor lysates. ELISA immunoassay was used to measure SDF1 protein levels in lysates of viable tumor from treated (N=4) and control (N=3) mice 72 h after partial thermal ablation. Data are expressed in pg per *μ*g of total protein and are presented as mean values ± standard deviation.

**Figure 4.**
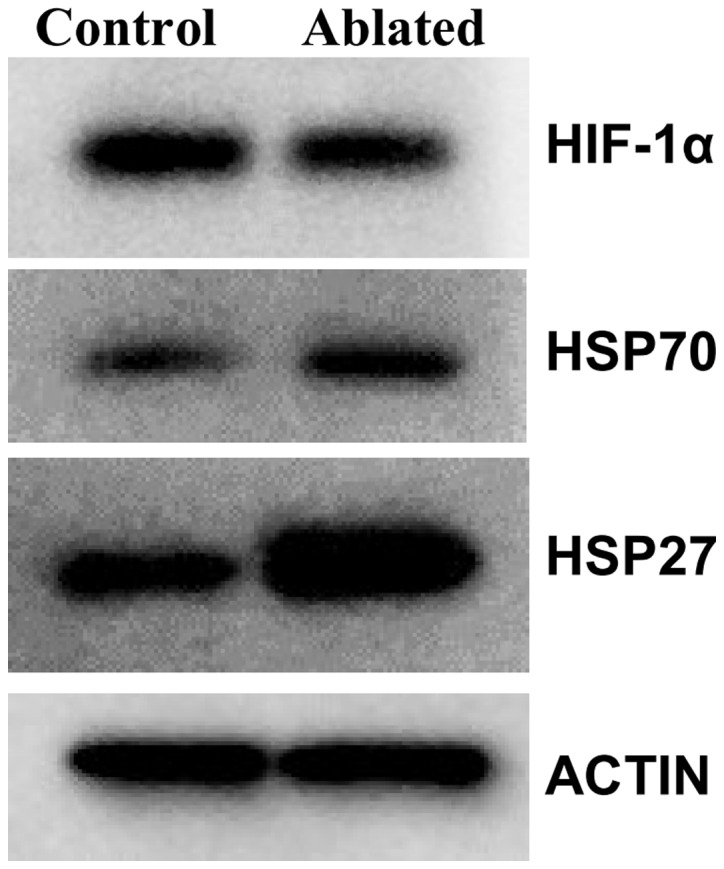
Protein levels for HIF1α and heat shock proteins in 4T1 tumor lysates. Western immunoblotting was used to measure protein levels of HIF1α, Hsp70 and Hsp27 in lysates of a viable tumor isolated from treated (N=5) and control (N=4) mice 72 h after partial thermal ablation. A representative blot is shown. Actin was used as the control protein.

The effects of partial tumor ablation on protein levels in viable tissue around the site of ablation 72 h after treatment were measured by immuno assays. As measured by ELISA, levels of SDF-1 protein in tumor lysates were significantly lower (0.071±0.016 pg/*μ*g versus 0.113±0.017 pg/*μ*g, P<0.05) for ablated (N=4) as compared to control (N=3) tumor tissue (Fig. 3). Levels of six proteins were measured in tumor lysates using western immuno blotting (Table II). A representative blot showing results for these proteins and actin, a control protein, are presented (Fig. 4). Levels of heat shock proteins HSP70 and HSP27 were higher for all individual samples for ablated (N=5) as compared to control (N=4) tumor tissue; whereas levels of hypoxia inducible factor (HIF-1α) were consistently lower for ablated tumors. Protein levels for matrix metallopeptidase-9 and antigen Ki-67 were unaffected by the treatment (data not shown).

### Effects on serum cytokine levels

**Figure 5.**
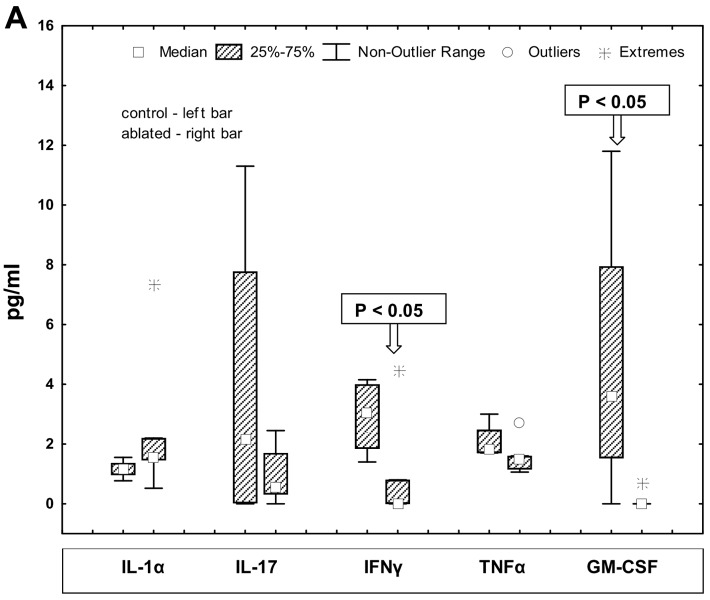

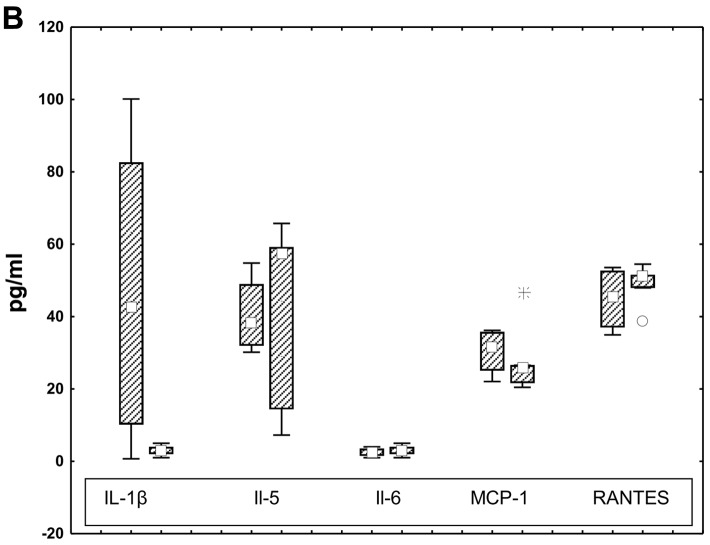
Cytokine levels in serum. Quansys multiplex immunoassay was used to measure 16 cytokines in serum isolated from treated (N=5) and control (N=4) mice 72 h after partial thermal ablation. Of the 16, 10 were present at detectable levels. (A) Cytokines present at up to 15 pg/ml. (B) Cytokines present at up to 100 pg/ml.

The effects of partial tumor ablation on serum cytokine levels 72 h after treatment were measured by multiplex immunoassay. The results for 10 of the 16 cytokines are presented (Fig. 5). The cytokines IL-1α, IL-17, IFNγ, TNFα and GM-CSF were present in serum at low concentrations. Other cytokines including IL-1β, IL-5, IL-6, MCP-1 and RANTES were present at higher concentrations. These ten cytokines were present at detectable levels in serum from at least five of the nine study mice and the inter-sample variability within groups was large. Only two cytokines, interferon-γ (IFNγ) and granulocyte-macrophage colony-stimulating factor (GM-CSF) showed significant differences between groups. IFNγ and GM-CSF had a significantly lower level in serum from the ablated mice as compared to control mice (P<0.05). Notably, interleukin-1β showed a trend towards lower values (P=0.154) in serum from ablated mice.

## Discussion

Thermal ablation has been used to treat tumors of the liver (20), prostate (21), and breast (22–24) in humans and animal models. Results of these therapies can vary greatly depending on many variables, including ablation modality, operator techniques and tumor site. For example, radiofrequency ablation can lead to accelerated perinecrotic outgrowth of colorectal liver metastases (25); whereas, we previously demonstrated that CITT therapy decreased metastasis in rabbits with VX2 tumors (6). In the current study, a mouse mammary carcinoma model was used to characterize the molecular changes that occur in serum, bone marrow, and tumor surviving partial ablation. At the transcriptional level, ablation increased *Sele, Fgf2, Lifr* and *Cxcl12* in marrow and decreased *Lifr* and *Sele* in the surviving tumor. At the protein level, ablation resulted in decreased levels of SDF1 and HIF-1α and increased levels of HSP27 and HSP70 in the surviving tumor. In serum, ablation decreased the concentration of the IFNγ and GM-CSF. Thus, 10 molecules have been identified that may be involved with alteration of communication between the marrow and tumor as well as alteration of homeostasis in surviving tumors.

The bone marrow responded to thermal ablation with an increase in the *Sele, Fgf2, Lifr* and *Cxcl12* transcripts. *Sele* encodes E-selectin, an adhesion molecule expressed by activated endothelial cells while tumor cells express E-selectin ligands. Interactions between these cell types through E-selectin are thought to regulate cancer metastases (26). Thus, the possibility exists that increased E-selectin could promote adhesion of cancer cells and enhance seeding tumor cells into the marrow. Fibroblasts growth factor 2 is a stromal cell mitogen and stimulates myelopoiesis in marrow. However, in the presence of advanced, untreated human breast and lung tumors, low levels of Fgf2 result in an arrest of maturation of mesenchymal stromal cells in marrow (27). The response of *Fgf2* to ablation seen here may be an attempt of the marrow to re-establish normal maturation for mesenchymal cells.

Leukemia inhibitory factor (LIF) is a cytokine that also affects marrow mesenchymal cells (28,29). Upregulation of the LIF receptor transcript *(Lifr)* in marrow may indicate that tumor ablation influences not only maturation but also differentiation of mesenchymal stromal cells. Stromal derived factor 1 is a signaling molecule for communication between tumor and marrow (30,31). The SDF1 receptor (CXCR4) is expressed on breast and other epithelial cancer cells and has been shown in multiple studies to be involved with metastasis (32–35). Increase of expression of the *Cxcl12* transcripts encoding SDF1 in bone marrow may stimulate mobilization and recruitement of immature hematopoietic cells, endothelial and smooth muscle progenitor to neo-angiogenic niche (36,37). However, it is unknown whether or not the elevation of *Cxcl12* transcript found in marrow post-ablation is evidence of seeding premetastatic niches or the primary tumor site. In summary, four transcripts have been identified which respond in marrow to tumor ablation. Further investigation is needed to confirm whether or not their encoded proteins play a role in mesenchymal cells development or tumor dissemination after thermal ablation.

Tumor tissue responded to thermal ablation with a decrease in SDF1 and HIF-1α proteins, and an increase in HSP27 and HSP70 proteins, and decreases in *Lifr* and *Sele* genes transcripts. Our work previously showed that thermal ablation decreases hypoxia in surviving tumor areas (7). The results presented here are consistent with this effect in that SDF1 and hypoxia inducible factor 1 (HIF-1α) are lower in treated than untreated tumors. These changes could inhibit the metastatic potential of surviving tumor cells or inhibit signaling between the marrow and tumor for the recruitment of cells supportive of tumor growth such as pro-angiogenic endothelial progenitor cells or marrow suppressor cells (32,35,38,39). The increase in heat shock proteins -70 and -27 after treatment may be a stress response. Unfortunately, HSP70 may be acting to protect the cancer cells as its expression has been associated with poor prognosis (40). However, HSP70 may also be indicative of a boost in antitumor immune activity post-ablation (41). Heightened tumor HSP27 is also associated with poor prognosis and metastasis (42) though evidence indicates multiple potential roles in tumorigenesis including promotion of tumor growth (43), regulation of epithelial-mesenchymal transition (44), and conferral of chemo-resistance (45). The increase of HSP27 implies that this heat shock protein should be closely monitored during thermal ablation of tumors and may be a useful marker to assist in determining appropriate treatment regimens in combination with thermal ablation. The effects of ablation on the *Sele* and *Lifr* genes were the opposite in tumor to that of marrow where we proposed that these factors may be involved with metastasis and mesenchymal cell development. It would be beneficial if the decrease seen in tumor was the result of a dampened potential for tumor cell proliferation and metastasis post-treatment. In summary, two transcripts and four proteins have been identified which respond in tumor tissue to ablation though additional pre-clinical studies are needed to define their functions and understand whether these molecules are involved with pro- or anti-tumorigenic responses after thermal ablation.

Serum analysis revealed that thermal ablation decreased concentrations of the granulocyte-macrophage stimulating factor (GM-CSF) and interferon-γ (IFNγ). These molecules function in inflammation and the immune response. Their levels are influenced both by the presence of solid tumors (46,47) and thermal ablation (48). However, the significance of their low levels post-ablation is unknown. These cytokines may be indicative of decreased need for antitumor immune function, immunosuppression by the treatment, or they may be associated with slowed tumor growth or smaller number of tumor cells after partial ablation (49,50). Future studies will be needed to determine the source, effects and meaning of low systemic levels of these cytokines three days after ablation therapy.

**Figure 6.**
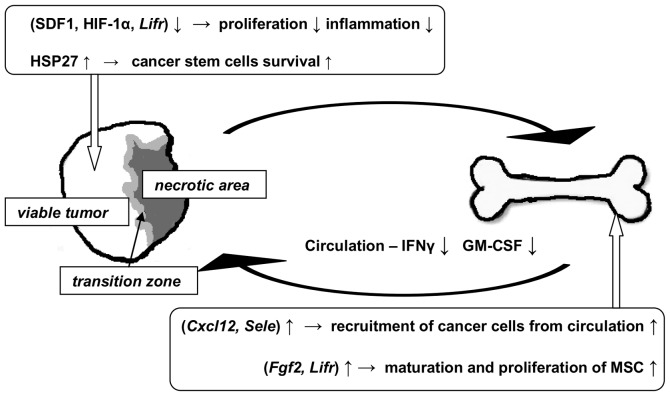
Summary results and hypothetical effects of molecular responses to tumor ablation. The molecular changes identified in this study after thermal ablation of mouse mammary carcinoma suggest decreased proliferation for surviving tumor cells and decreased local and systemic inflammation. These changes are accompanied by molecular evidence for increased maturation and proliferation of bone marrow cells and recruitment of circulatory cancer cells to the marrow. Elevated HSP27 in the perinecrotic area may be a marker of a robust population of cancer stem cells that have survived tumor ablation.

The major findings and proposed implications of the study are summarized in Fig. 6. Thermal ablation appears to decrease local and systemic decrease of inflammation. Proliferation of viable tumor cells may decrease but HSP27 may indicate the presence of a protected population of surviving cancer cells that may be responsible for aggressive tumor re-growth. Additionally, thermal ablation caused upregulation of genes in bone marrow that are associated with maturation and proliferation of mesenchymal stem and hematopoietic cells to possibly replenish immune cells used during the initial response to trauma. The increase of gene expression encoding SDF1, a ligand for cancer cells, could increase the risk for circulating cancer cells to establish themselves in marrow. We conclude that thermal ablation clearly impacts transcript and protein levels of molecules that may be involved in pro- and anti-tumor activity in the tumor itself, in bone marrow and in serum. However, additional studies are needed to determine the influence of these changes on cell phenotype and their significance relative to cancer host or patient outcomes.

## Figures and Tables

**Table I.** The genes analyzed in bone marrow and 4T1 tumors from BALB/c mice.

Symbol	Molecule name	Description (function in breast cancer)
Assay type: Quantitative RT-PCR in bone marrow and tumor lysates		
*Bmp2*	Bone morphogenic protein 2	Growth factor, TGFβ superfamily - tumor apoptosis, EMT
*Ccl2*	Chemokine (C-C motif) ligand 2 (MCP-1)	Signaling molecule - TAM infiltration, cancer stem cell
*Csf3*	Colony stimulating factor 3 (granulocyte) (G-CSF)	Cytokine-apoptosis, neutrophils release from BM
*Csf3r*	Colony stimulating factor 3 receptor (granulocyte)	CD114, hematopoietin receptor family - cell maturation in BM
*Cxcl12*	Chemokine (C-X-C motif) ligand 12 (SDF-1)	Signaling molecule - hematopoietic cell homing and quiescence, communication between tumor and BM
*Cxcr4*	Chemokine (C-X-C motif) receptor 4 (SDF-1 receptor)	CD184 (fusin), α-chemokine receptor - metastasis to BM
*Fgf2*	Fibroblast growth factor 2	Growth factor (basicFGF) - myelopoiesis and angiogenesis in BM
*Glg1*	Golgi apparatus protein 1 (ESL-1)	Glycoprotein, leukocyte ligand for E-selectin-metastasis
*Hsf1*	Heat shock factor 1	Nuclear protein activating HSPs-growth, inhibition of apoptosis
*Icam1*	Intercellular adhesion molecule 1	CD54, glycoprotein, immunoglobulin family-metastasis
*Id1*	Inhibitor of DNA binding 1	Nuclear protein - metastasis, inhibition of differentiation
*Il6*	Interleukin 6	Pleiotropic cytokine - metastasis to BM
*Il17ra*	Interleukin 17 receptor A	CD217, glycoprotein - proinflamatory, associated with poor prognosis
*Kdr*	Kinase insert domain protein receptor (VGFR2)	VGEF receptor - angiogenesis, tumor progression
*Klrk1*	Killer cell lectin-like receptor subfamily number 1	CD314 (NKG2D), NK cell receptor-antitumor immunity
*Lif*	Leukemia inhibitory factor	Pleiotropic cytokine-tumor proliferation, chemoattractant for BM derived cells
*Lifr*	Leukemia inhibitory factor receptor	CD118, cytokine receptor - suppressor of metastasis
*Mmp2*	Matrix metallopeptidase 2	Type IV collagenase, gelatinase A - metastasis
*Mmp9*	Matrix metallopeptidase 9	Type IV collagenase, gelatinase A - angiogenesis, metastasis
*Ptgs2*	Prostaglandin-endoperoxide synthase 2	Cyclooxygenase 2 (COX2) enzyme - worse prognosis
*Sele*	E-selectin	CD62, adhesion molecule, expressed only on activated endothelial cells - metastasis
*Serpin1*	Serine protease inhibitor, nectin (PAI-1)	Inhibitor of tissue plasminogen activator (tPA) - cancer progression
*Tnfrsf11a*	Tumor necrosis factor receptor superfamily, member 11a (RANK)	Expressed on osteoclasts and dendritic cells - bone metastasis
*Tnfsf11*	Tumor necrosis factor (ligand) superfamily, member 11 (RANKL)	CD254, expressed on osteoblasts, stromal and T cells - cancer cells chemoattractant
*Tnf*	Tumor necrosis factor	Cachectin, TNFα-cytotoxin - breast cancer promoter
*Ucp2*	Uncoupling protein 2 (mitochondrial proton carrier)	Mitochondrial ROS regulator - tumor promoting factor
*Vcam1*	Vascular cell adhesion molecule 1	CD106, mediator of immune cell adhesion to endothelium - aberrantly expressed on breast cancer cells, metastasis

**Table II.** List of proteins analyzed in 4T1 tumors and serum from BALB/c mice.

Symbol	Molecule name	Description (function in breast cancer)
Assay type: ELISA in tumor lysates		
CXCL12	Chemokine (C-X-C motif) ligand 12 (SDF-1)	Signaling molecule - hematopoietic cell homing and quiescence, communication between tumor and BM
Assay type: Western immunoblotting in tumor lysates		
HIF-1α	Hypoxia-inducible factor 1A	Transcription factor - adaptation to hypoxia, overexpression in invasive cancers
HSP27	Heat shock protein 27	Stress response protein - drug resistance, stem cell maintenance
HSP70	Heat shock protein 70	Stress response protein - antigen binding, anti-apoptotic
MMP9	Matrix metallopeptidase 9	Type IV collagenase, gelatinase A - angiogenesis, metastasis
Ki67	Antigen Ki-67	Nuclear protein - marker of proliferation
Assay type: Antibodies multiplex of 16 murine chemokines in serum		
IL-1α, IL-1β, IL-2, IL-3, IL-4, IL-5, IL-6, IL-10, IL-12, Il-17, MCP-1, IFNγ, TNFα, MIP-1α, GM-CSF, RANTES		Signaling molecules with immunomodulatory and chemotaxic functions - altered in serum of breast cancer patients

**Table III.** The murine primers used.

Gene	Accession no.	Forward primer	Reverse primer
*Bmp2*	NM_007553	TGTCCCCAGTGACGAGTTTCT	CCTGTATCTGTTCCCGGAAGAT
*Ccl2*	NM_011333	CTGAAGCCAGCTCTCTCTTCCT	CAGGCCCAGAAGCATGACA
*Csf3*	NM_009971	CAGTACCCCCAAAAAATCAGTGA	TGGGCCCCCCTGAGAT
*Csf3r*	NM_007782	TCCAGCGAGTCCCCAAAG	CAGCATGGGAGGCTCCAAT
*Cxcl12*	NM_021704	GCCTCCAAACGCATGCTT	ATTGGTCCGTCAGGCTACAGA
*Cxcr4*	NM_009911	TCGGCAATGGATTGGTGAT	CCGTCATGCTCCTTAGCTTCTT
*Fgf2*	NM_008006	TGGTATGTGGCACTGAAACGA	TCCAGGTCCCGTTTTGGAT
*Glg1*	NM_009149	CTCACTGCGCCCTCTAACG	GGCACCTGATGCTGCTCTACT
*Hsf1*	NM_008296	CATAAAAATACGCCAGGACAGTGT	CCCCTTCATCAGCTGCACAT
*Icam1*	NM_010493	TGGCGGGAAAGTTCCTGTT	TCCAGCCGAGGACCATACA
*Id1*	NM_010495	GAACGTCCTGCTCTACGACATG	TGGGCACCAGCTCCTTGA
*Il-17ra*	NM_008359	CCCAGGCAAGAAGAATTCCA	CACCAGTGAAACTTGCTTAGAGTGA
*Il-6*	NM_031168	CCACGGCCTTCCCTACTTC	TTGGGAGTGGTATCCTCTGTGA
*Kdr*	NM_010612	ACTGCAGTGATTGCCATGTTCT	TCATTGGCCCGCTTAACG
*Klrk1*	NM_033078	GGCAATTCGATTCACCCTTAAC	ATACTGGCTGAAACGTCTCTTTGA
*Lif*	NM_008501	GCCACGGCAACCTCATG	ATTGGCGCTGCCATTGA
*Lifr*	NM_013584	AGAACATCACTGACATATCCCAGAAG	GTATAGGCTCGCAGGACCAGAT
*Mmp2*	NM_008610	GGACCCCGGTTTCCCTAA	CAGGTTATCAGGGATGGCATTC
*Mmp9*	NM_013599	TGGTGGCAGCGCACG	CTTCCGGCACGCTGGA
*Ptgs2*	NM_011198	TGCCTCCCACTCCAGACTAGA	CAGCTCAGTTGAACGCCTTTT
*Sele*	NM_011345	TCCTGCGAAGAAGGATTTGAA	CCCCTCTTGGACCACACTGA
*Serpin1*	NM_008871	CCGTGGAACAAGAATGAGATCAG	CTCTAGGTCCCGCTGGACAA
*Tgfb1*	NM_011577	GCAGTGGCTGAACCAAGGA	AGCAGTGAGCGCTGAATCG
*Tnf*	NM_013693	CACAAGATGCTGGGACAGTGA	TCCTTGATGGTGGTGCATGA
*Tnfrsf11a*	NM_009399	TCGTCCACAGACAAATGCAAA	GTGTGCTTCTAGCTTTCCAAGGA
*Tnfsf11*	NM_011613	GGCCACAGCGCTTCTCA	CCTCGCTGGGCCACATC
*Ucp2*	NM_011671	GCCCCTTCACCTCTTTAGCA	CCAAGCACTGGGAAGGTCTAAC
*Vcam1*	NM_011693	CTCCCCTGAATACAAAACGATTG	GCCCGTAGTGCTGCAAGTG

## Abbreviations:

CITT: conductive interstitial thermal therapy
